# Correlation Analysis Between Serum Uric Acid, Prealbumin Level, Lactate Dehydrogenase, and Severity of COVID-19

**DOI:** 10.3389/fmolb.2021.615837

**Published:** 2021-07-12

**Authors:** Zhenmu Jin, Mo Zheng, Jichan Shi, Xinchun Ye, Fang Cheng, Que-Lu Chen, Jianping Huang, Xian-Gao Jiang

**Affiliations:** ^1^ Department of Rheumatology and Immunology and Allergy, Wenzhou Central Hospital Medical Group, The Dingli Clinical Institute of Wenzhou Medical University, Wenzhou, China; ^2^ Department of Infectious Diseases, Wenzhou Central Hospital Medical Group, The Dingli Clinical Institute of Wenzhou Medical University, Wenzhou, China; ^3^ Department of Radiology and Imaging, Wenzhou Central Hospital Medical Group, School of Clinical Theory, Wenzhou Medical University, Wenzhou, China; ^4^ Department of Neurology, Wenzhou Central Hospital, The Second Affiliated Hospital of Shanghai University, Affiliated Dingli Clinical Institute of Wenzhou Medical University, Wenzhou, China

**Keywords:** correlation analysis, COVID-19, LDH, prealbumin, serum uric acid

## Abstract

**Objective:** To analyze the correlation between serum uric acid, prealbumin levels, lactate dehydrogenase (LDH), and the severity of COVID-19.

**Methods:** The data from 135 patients with COVID-19 was collected, and the patients were divided into a non-severe group (110 cases) and a severe group (25 cases), according to the severity of illness. Sixty cases with normal physical examinations over the same period and 17 cases diagnosed with other viral pneumonia in the past five years were selected as the control group to analyze the correlation between the detection index and the severity of COVID-19.

**Results:** Serum albumin and prealbumin in the severe group were significantly lower than those in the non-severe group (*p* < 0.01); serum uric acid in the severe group was lower than that in the non-severe group (*p* < 0.05). LDH and C-reaction protein (CRP) in the severe group were higher than those in non-severe group (*p* < 0.01); the levels of albumin, prealbumin, serum uric acid, and LDH in the severe group were significantly different from those in healthy control group (*p* < 0.01) and the levels of prealbumin, serum uric acid, LDH, and CRP in the severe group were significantly different from those in the other viral pneumonia group (*p* < 0.01). Serum albumin and prealbumin were positively correlated with the oxygenation index (*p* < 0.001), while LDH was negatively correlated with oxygenation index (*p* < 0.001).

**Conclusion:** Serum albumin, prealbumin, the oxygenation index, and LDH are risk factors of COVID-19.

## Introduction

COVID-19 is an infectious disease mainly spread by droplets**.** It spreads quickly and is a severe disease that poses a great threat to people's health (Kahn et al., 2020; Kim et al., 2020; Kiss et al., 2020; Lim and Claydon, 2020). As this is a recently discovered disease, there is no conclusion on its pathogenesis and risk factors (Djulbegovic et al., 2020). Therefore, it is very important to study the changes and clinical significance of various indexes of patients to understand the disease, drug development, and treatment of patients (O'Reilly et al., 2020). COVID-19 and SARS are both coronaviruses, so we can speculate that the two may have similarities in some respects (Abdollahimajd et al., 2020; Chang and Lipner, 2020; Okudela et al., 2020; Pakos et al., 2020).

The main organ affected by COVID-19 is the lungs, which suffer diffuse inflammation and the inflammatory storms that impair and cause lung damage in patients. In lung infections, melatonin has the ability to act as a potent antioxidant and anti-inflammatory agent and has key roles in the mitigation of oxidative stress and the excessive production of pro-inflammatory cytokines and chemokines in lung tissues. Solomon Habtemariam et al. recommended more basic and clinical studies on the anti-infective effects of melatonin against different types of lung infections (Habtemariam et al., 2016).

Previous literature has shown that liver function involvement is found in both SARS and MERS patients, and hypoalbuminemia is significantly positively correlated with the poor prognosis of the disease (Vecchié et al., 2020). For every 10 g/L decrease in serum albumin concentration, the mortality rate increases significantly by 137 and the morbidity rate increases by 89 (Alser et al., 2020). In addition, patients with severe COVID-19 may have other organ dysfunctions besides respiratory system involvement (Mattioli et al., 2020). Therefore, it also suggests that, for such patients, early identification of other organ dysfunction is particularly important. Previous studies have confirmed that patients with COVID-19 can have abnormal liver function, which is manifested by increased ALT and AST levels and decreased albumin levels, but there are fewer reports of abnormal levels of other indexes (Grazzi et al., 2020).

Therefore, this study collected data of patients with COVID-19 and divided them into non-severe groups and severe groups according to their severity. Healthy people with normal physical examinations in the same period and cases diagnosed with other viral pneumonia in the past five years were selected as the control group to analyze the correlation between the detection index and the severity of COVID-19.

## Methods

### Research Object and Sampling Size

The data of 135 patients diagnosed with COVID-19 in our hospital from January 21, 2020, to February 29, 2020, were collected and retrospectively analyzed. The diagnostic criteria for COVID-19 are in line with *Diagnosis and Treatment Plan for COVID-19 (Trial Version 5, Revised Edition)* by the National Health Commission (Kim et al., 2020). Sixty cases with normal physical examinations in the same period and 17 cases diagnosed with other viral pneumonia in the past five years were selected as the control group. This study focuses on analyzing the risk factors of severe new coronavirus pneumonia. Therefore, other viral pneumonias are selected to match severe COVID-19 pneumonia at a 1:1 ratio in sample size. When we selected patients for this study, because other viral pneumonias are more common in children and relatively rare in adults, and because fewer patients were diagnosed with COVID-19 pneumonia during this study, after matching for gender and age, only 17 cases were used as controls. In the healthy control group, according to a 1:2 ratio matching, 60 patients were actually selected as controls. In terms of gender and age, there are no significant differences among other viral pneumonias, healthy controls, and severe COVID-19 pneumonia. This study was approved by the Ethics Committee of Wenzhou Central Hospital, and informed consent was obtained from patients and their families.

### Case Data Collection

The following data was collected by reviewing the case data: 1) general data such as gender, age, smoking history, epidemiological contact history, and comorbidities. The epidemiological history includes travel to Wuhan or close contact with patients in Wuhan two weeks before the onset of illness, and travel to the gathering place (Yintai Shopping Center; 2) Viral nucleic acid detection using nasopharyngeal swab or sputum sampling and real-time fluorescence quantitative reverse transcription-PCR method. Fasting blood routine tests, blood biochemistry, CRP, and erythrocyte sedimentation rate (ESR) were collected from 7:00am to 8:00am on the second day of admission after 8–10 h of fasting. 4) According to the relevant clinical classification standards of *Diagnosis and Treatment Plan for COVID-19 (Trial Version 5, Revised Edition)* by the National Health Commission, the cases are divided into mild, common, severe, and critical. The mild and common types were assigned to the non-severe group, and the severe and critical types are divided into the severe group.

### Observation Index

The general data (including age, gender, epidemiological history, complications, and clinical characteristics) and detection indexes (serum uric acid, albumin, prealbumin, LDH, oxygenation index, etc.) of the three groups were compared.

### Statistical Process

SPSS 24.0 was used for statistical analysis. The measurement data of normal distribution was x ± s, and the count data was expressed as a percentage. Multi-group comparison was performed by a single-factor analysis of variance. Correlation analysis was performed by the Spearman correlation analysis. Regression analysis was performed by multiple logistic regression analysis, where *p* < 0.05 is considered statistically significant.

## Results

### Comparison of General Data

As shown in Table 1, the basic data of each group had no significant statistical difference (*p* > 0.05), indicating that the data of each group was comparable.

**TABLE 1:** General_information_of_135_novel_coronavirus_infected_pneumonia_patients.

Program	Patients (N = 135)	Disease severity		*p*
		Not serious (*n* = 110)	serious (*n* = 25)	
Age (year)				
19–30	22	20	2	*p* > 0.05
31–60	97	82	15	
＞61	16	8	8	
Female	64	57	7	*p* > 0.05
Complication				
hypertension	30	22	8	*p* > 0.05
diabetes	6	5	1	
Chronic lung disease	3	3	0	
coronary Heart disease	3	1	2	
tumor	4	2	2	
Chronic liver disease	13	10	3	
Connective tissue disease	1	1	0	
gout	3	2	1	
Clinical symptoms				*p* > 0.05
Fever	107	85	22	
Fear of cold	31	20	11	
tosse	80	61	19	
Expectoration	50	34	16	
weakness	20	15	5	
Chest pain	4	2	2	
Myalgia	10	8	2	
headache	6	5	1	
sore Throat	16	14	2	
Shortness of breath	15	7	8	
diarrhea	14	12	2	

### Comparison of Detection Indexes

As shown in Table 2, the levels of serum albumin, prealbumin, LDH, uric acid, and C-reactive protein in the severe group were significantly different from those in the non-severe group. Serum albumin and prealbumin in the severe group were significantly lower than those in the non-severe group (*p* < 0.01); serum uric acid in the severe group was lower than that in the non-severe group (*p* < 0.05). The levels of LDH and CRP in the severe group were higher those in the non-severe group (*p* < 0.01); the levels of albumin, prealbumin, serum uric acid, and LDH were significantly different between the severe group and the healthy control group (*p* < 0.01) and the levels of prealbumin, serum uric acid, LDH, and CRP in the severe group were significantly different from those in the other viral pneumonia groups (*p* < 0.01).

**TABLE 2:** Comparison_of_four_groups_of_detection_index_level.

Group	ALT	AST	Serum albumin (g/L)	Serum prealbumin (mg/L)	Serum uric acid (μmol/L)	Serum lactate dehydrogenase (U/L)	C-reactive protein (mg/L)
Covid-19 non severe group (n = 110)	29.8 ± 30.1	28.0 ± 17.6	42.6 ± 3.6*	197.1 ± 48.4Δ*	241.4 ± 90.9Δ*	201.0 ± 61.9Δ*	15.0 ± 19.6Δ
Covid-19 severe group (n = 25)	36.8 ± 25.3	42.9 ± 25.8	37.6 ± 3.5Δ	119.9 ± 45.0Δ	197.8 ± 97.1Δ	296.0 ± 68.0Δ	50.7 ± 28.8Δ
Non covid-19 (n = 17)	70.8 ± 47.9	55.5 ± 36.5	37.9 ± 5.2Δ	175.5 ± 41.5Δ	308.7 ± 114.3	410.8 ± 252.8Δ	20.6 ± 16.5Δ
Healthy Control group (n = 60)	21.4 ± 13.8	22.7 ± 6.4	42.5 ± 3.5	246.0 ± 61.1	291.8 ± 68.0	177.6 ± 30.2Δ	1.4 ± 1.6
F value	14.3	17.7	18.7	35.5	10.0	39.1	45.4
*p* value	0.000	0.000	0.000	0.000	0.000	0.000	0.000

ΔCompared with the healthy control group p ＜ 0.001,*Compared with covid-19 severe group, p ＜ 0.001.

### Correlation Analysis Between the Oxygenation Index and Serum Albumin, Prealbumin, and LDH

**FIGURE 1:**
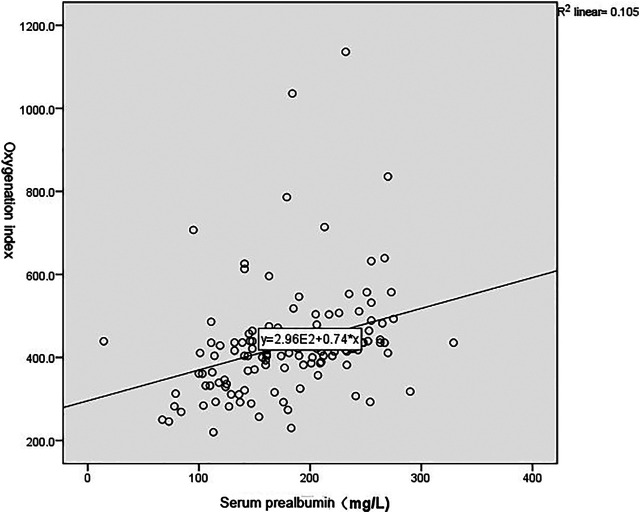
Correlation between serum prealbumin and oxygenation index.

**FIGURE 2:**
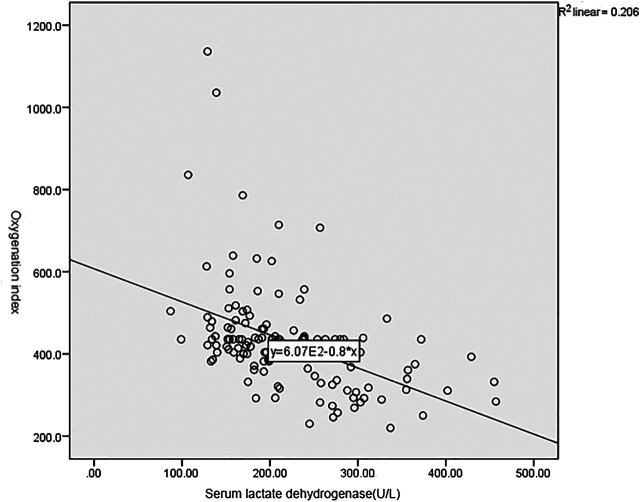
Correlation between LDH and oxygenation index.

As shown in Figure 1, Figure 2, and Table 3, the Pearson correlation analysis showed that serum albumin and prealbumin were positively correlated with the oxygenation index (*p* < 0.001), while LDH was negatively correlated with the oxygenation index (*p* < 0.001).

**TABLE 3:** Correlation_analysis_of_serum_albumin, _prealbumin, lactate_dehydrogenase_and_oxygenation_index.

Index	Serum albumin		Serum prealbumin		Lactate dehydrogenase	
	R value	*p* value	R value	*p* value	R value	*p* value
Oxygenation index	0.445	0.000	0.436	0.000	−0.522	0.000

### Analysis of Risk Factors

After multi-factor logistic regression analysis, a total of three quantitative indexes entered the final model: the oxygenation index and the level of serum albumin, prealbumin, and LDH Table 4.

**TABLE 4:** Multivariate logistic regression analysis of covid-19 clinical screening.

Index	regression coefficient	Wald χ2	P	OR	95% Confidence interval
serum albumin	−0.227	4.844	0.028	0.797	0.651∼0.975
Prealbumin	−0.027	7.448	0.006	0.974	0.955∼0.993
Oxygenation index	−0.018	8.861	0.003	0.982	0.97∼0.994

## Discussion

This study showed that compared to the healthy control group, the level of serum albumin, prealbumin, and serum uric acid in the severe group was significantly lower; the albumin level of the non-severe group had no statistically significant difference compared with the non-severe group and the serum albumin and prealbumin in the severe group were significantly lower, suggesting that serum prealbumin may be a sensitive index of the severity of the disease.

A meta-analysis showed that hypoalbuminemia was a strong, dose-dependent independent predictor of poor prognosis (Montero et al., 2020). For every 10 g/L decrease in serum albumin concentration, the mortality rate increases by 137, the morbidity rate increases by 89, and the length of stay in the intensive care unit and hospital stay increases 28 and 71% respectively (Santosh, 2020). Studies have shown that, in addition to respiratory system involvement, patients with severe COVID-19 can also experience other organ dysfunction, and even multiple organ failure, leading to death (Salepci et al., 2020). Early recognition of the involvement of other organ functions is particularly important. Many studies have confirmed that patients with COVID-19 can have abnormal liver function, which is manifested by increased ALT and AST levels, decreased albumin levels, and increased TBil levels in a few cases. High levels of AST, ALT, ALP, and GGT may be due to the escape of these enzymes from the liver cytosol into the blood stream. And the liver biosynthesis dysfunction and defect of these enzymes are always accompanied by the changes of liver membrane permeability. A study showed that propolis could reduce AST, ALT, ALP, and GGT activities in rats which could prove to be a potential therapy treatment to patients (Talas et al., 2013).

There are a few reports of abnormal prealbumin and serum uric acid levels (Salepci et al., 2020). It has been reported that prealbumin is superior to albumin in the diagnosis of VAP, which is helpful for early diagnosis of VAP and active intervention and treatment (Salepci et al., 2020). Chen et al. found that 99 cases of COVID-19 patients accounted for 98 cases with abnormal albumin levels. Involvement of liver function has been reported in patients with SARS and MERS. Prealbumin PA is a serum protein synthesized by liver cells. Its main physiological function is to transport thyroxine and vitamin A, and it has thymic hormone activity. It can enhance the immunity of the body by promoting the maturation of lymphocytes. The molecular weight of PA is 54,980, and the electrophoresis rate of PA is 25%, which is higher than that of albumin in agar electrophoresis. PA is in front of albumin, hence the name. Serum PA is one of the fast transporters produced by hepatocytes. Its daily decomposition rate is 33.1–39.5%, and its half-life is 1.9 D. This index can reflect the slight changes of liver synthesis and catabolism, and the extent of serum concentration reduction is closely related to the degree of liver parenchymal damage. As a negative acute phase protein, PA is a non-specific host defense substance, which can remove toxic generation products released in the blood circulation during infection. In the process of acute phase reaction, the PA level decreases rapidly. In SARS patients, PA decreased significantly compared with healthy people and other pneumonia patients.

LDH is widely found in cardiac muscle, skeletal muscle, liver, kidney, brain, and other tissue cells. When these tissue cells suffer from ischemia, hypoxia, edema, and other injuries, LDH can be released into the blood. This study also found that LDH levels in both mild and severe patients with COVID-19 were significantly higher than those in healthy controls, suggesting that LDH was changed in the early stage of COVID-19 patients. Various literature has reported that LDH levels in acute and convalescent patients with severe viral pneumonia were higher than that in non-severe patients. This may be related to lung inflammation and hypoxia, especially when accompanied by lung interstitial damage. And Ozdemir Ilknur et al. found that in DMBA-induced increased LDH rats model, two kinds of organoselenium compounds, Sel and Sell, caused a significant decrease in LDH activities, and these organoselenium compounds are likely to be beneficial in patient health (Ozdemir et al., 2007).

## Conclusion

Serum albumin, prealbumin, oxygenation index, and LDH were risk factors of COVID-19.

## Data Availability

The datasets presented in this study can be found in online repositories. The names of the repository/repositories and accession number(s) can be found in the article/Supplementary Material.
